# Post-Stroke Recrudescence During the Perioperative Period: Potential Roles of Anesthetic and Sedative Agents

**DOI:** 10.7759/cureus.114851

**Published:** 2026-08-20

**Authors:** Mahdi Fadel, Ali Fadel, Nataliah Jwaida, Adam Berry, Marwan Shuayto

**Affiliations:** 1 Medicine, American University of the Caribbean School of Medicine, Cupecoy, SXM; 2 Medicine, Wayne State University, Detriot, USA; 3 Medicine, Wayne State University School of Medicine, Detroit, USA; 4 Medicine, Wayne State University, Detroit, USA; 5 Neurology, Michigan Neurology and Spine Center, Port Huron, USA

**Keywords:** anesthetic and sedative agents, postoperative neurologic deficit, post-stroke recrudescence, stroke mimics, stroke recrudescence

## Abstract

Post-stroke recrudescence (PSR) is the transient reappearance of previously recovered focal neurologic deficits after a systemic or pharmacologic stressor, without evidence of a new cerebral infarction. In the perioperative setting, PSR can closely resemble acute ischemic stroke, residual anesthetic effects, seizure, or delirium, creating a significant diagnostic challenge. This narrative review examines the proposed mechanisms of PSR and evaluates the available evidence regarding anesthetic, sedative, and analgesic agents as potential triggers. Previously injured neural networks may remain vulnerable to disruptions in excitatory-inhibitory balance despite apparent clinical recovery. Human evidence is strongest for midazolam and broader benzodiazepine exposure, while evidence involving opioids, propofol, volatile anesthetics, and dexmedetomidine remains limited or indirect. Determining the independent effect of any single medication is difficult because surgery frequently involves simultaneous stressors, including hypotension, hypoxemia, infection, electrolyte abnormalities, pain, and sleep disruption. PSR typically reproduces the pattern of a patient’s prior stroke deficits, is not associated with new diffusion-weighted imaging abnormalities, and improves after correction of the underlying trigger or resolution of medication effects. However, PSR remains a diagnosis of exclusion, and its consideration should not delay prompt evaluation for acute stroke. Recognition may be improved through clear preoperative documentation of baseline neurologic findings, comparison of postoperative deficits with the previous stroke syndrome, and early communication among anesthesiology, surgical, and neurology teams. Current evidence does not establish most anesthetic agents as independent causes of PSR, highlighting the need for prospective studies that document specific agents, doses, timing, concurrent perioperative stressors, and neurologic outcomes.

## Introduction and background

Post-stroke recrudescence (PSR) describes the transient reappearance of prior focal neurologic deficits in patients with remote stroke, provoked by systemic physiologic stressors rather than new brain infarction [1]. Data suggest recrudescence occurs a mean of 3.9 years after the index stroke [1]. Clinically, episodes last a mean of 18.4 hours in 69% of cases; deficits are typically abrupt and mild, with a mean National Institutes of Health Stroke Scale (NIHSS) worsening of 2.5 points [1,2].

Unlike acute ischemic stroke, PSR is a transient reemergence or worsening of previously experienced deficits from an old established stroke without evidence of a new infarction on diffusion-weighted imaging and typically resolves after correction of the precipitating stressor [1]. Conversely, acute ischemic stroke is a new event caused by fresh vascular occlusion causing new tissue injury. Distinguishing them is critical because their management diverges sharply. PSR centers on treating the provoking factor, whereas acute ischemic stroke requires immediate imaging and reperfusion evaluation to preserve salvageable tissue [3].

In the perioperative period, PSR poses a particular diagnostic challenge. Residual anesthetic and analgesic effects can mask or mimic new focal findings, while common intraoperative stressors - hypotension, hypoxemia, and metabolic shifts - further compromise marginally functioning peri-infarct networks. Benzodiazepines and opioids have been repeatedly associated as triggers, potentially through enhanced tonic GABAergic inhibition and opioid-mediated suppression of vulnerable cortical regions [1,4].

Yet no stroke assessment scale, including commonly used tools such as the NIHSS, has been specifically validated for detecting acute neurologic changes in the perioperative setting [2]. The NIHSS is a standardized clinical tool used to quantify neurologic impairment by assessing domains such as level of consciousness, motor function, sensation, language, speech, and neglect, with higher scores generally indicating greater neurologic deficit. Additionally, no standardized screening protocol exists to identify at-risk patients with prior cerebrovascular disease or surgical patients at risk for PSR [3].

This focused narrative review examines the pathophysiological mechanisms and perioperative triggers of PSR, with particular attention to the role of anesthetic and analgesic agents. It further addresses the diagnostic challenge of distinguishing recrudescence from acute ischemic stroke and proposes a framework to reduce the clinical consequences of misdiagnosis in this underrecognized population.

## Review

Methodology

Review Design

This study was conducted as a focused narrative review examining the pathophysiology, clinical evidence, and potential association of anesthetic, sedative, and analgesic agents with post-stroke recrudescence (PSR) in the perioperative setting. A narrative approach was selected because the available literature is limited and heterogeneous, encompassing mechanistic and preclinical studies, prospective challenge studies, retrospective observational studies, and reviews.

Data Sources

Relevant literature was identified through searches of PubMed and Google Scholar, supplemented by OpenEvidence, an AI-assisted medical literature retrieval tool, and manual review of the reference lists of pertinent publications. Searches were conducted through August 2026.

Search Terms

Searches used combinations of terms including “post-stroke recrudescence,” “recrudescence of stroke deficits,” “stroke mimic,” “reemergence of neurologic deficits,” “unmasking of neurologic deficits,” “peri-infarct cortex,” “anesthesia,” “anesthetic,” “sedation,” “sedative,” “benzodiazepine,” “midazolam,” “opioid,” “fentanyl,” “propofol,” “dexmedetomidine,” “volatile anesthetic,” “GABA,” “perioperative stroke,” “postoperative neurologic deficit,” “postoperative delirium,” “cognitive bias,” and “inter-specialty communication.” English-language sources relevant to PSR epidemiology and clinical course, proposed pathophysiologic mechanisms, pharmacologic associations, perioperative neurologic assessment, differential diagnosis, and factors contributing to diagnostic error were considered.

Source Selection

Sources were selected based on their relevance and contribution to the principal themes of the review. Literature considered included original clinical studies, preclinical and mechanistic studies, systematic and narrative reviews, and professional society scientific statements or clinical guidance.

Evidence Appraisal

Evidence was qualitatively assessed according to study design, sample size, presence of a control or comparator, potential confounding factors, and directness of the evidence to PSR. Particular consideration was given to concomitant perioperative stressors, including infection or systemic inflammation, hypotension, hypoxemia, electrolyte abnormalities, pain, and sleep disruption, because these factors may complicate attribution of neurologic deterioration to an individual pharmacologic agent. Any descriptions of evidence strength used in this review are qualitative and do not represent a formal validated evidence-grading or risk-of-bias framework.

Data Synthesis

Findings were synthesized narratively and organized according to proposed pathophysiologic mechanisms, individual pharmacologic agents or classes, the multifactorial effects of general anesthesia and concomitant perioperative stressors, and the clinical differentiation of PSR from acute ischemic stroke and other postoperative neurologic mimics. Literature addressing cognitive bias, interdisciplinary communication, and other contributors to diagnostic error was additionally incorporated into the discussion of perioperative diagnostic challenges. Key characteristics and findings from the principal studies, together with the distinguishing clinical, radiologic, and electroencephalographic features of PSR and its principal perioperative mimics, were summarized to facilitate comparison.

Limitations of the Review Methodology

Because this review was narrative in scope, formal quantitative synthesis, meta-analysis, and risk-of-bias scoring were not performed. The available literature spans heterogeneous study designs, patient populations, pharmacologic exposures, and clinical outcomes that are not readily suited to quantitative pooling. Instead, the review aimed to synthesize the available clinical and mechanistic evidence, evaluate the biologic plausibility and strength of reported associations between anesthetic, sedative, and analgesic agents and PSR, identify important perioperative confounding factors, and highlight diagnostic considerations and remaining knowledge gaps.

Pathophysiology and proposed mechanisms

A proposed pathophysiological mechanism of PSR involves disruption of compensatory neuroplasticity within the peri-infarct zone, where surviving neurons maintain a fragile excitatory-inhibitory balance that is readily overwhelmed by metabolic or physiologic stressors such as hyperthermia, infection, metabolic derangement, or systemic inflammation [1]. This vulnerability is rooted in a chronic pathological shift in GABAergic tone: after stroke, tonic inhibition mediated by extrasynaptic α5- and δ-subunit-containing GABA(A) receptors is upregulated in peri-infarct cortex, driven by impaired GABA transporter (GAT-3/GAT-4) function and elevated ambient GABA concentrations [4]. This excessive tonic inhibition suppresses low-gamma oscillations - neural rhythms critical for coordinated plasticity during the recovery window - thereby constraining the capacity for functional remapping [5]. 

Pharmacologic agents commonly administered during anesthesia further depress these already compromised networks. Benzodiazepines with broad subunit affinity enhance both tonic and phasic GABAergic currents, but their detrimental impact on post-stroke circuits appears driven primarily by augmentation of the pathologically elevated tonic component rather than phasic (synaptic) inhibition, which may instead support recovery [6]. Opioids compound this vulnerability through μ-receptor-mediated presynaptic inhibition of glutamate release and postsynaptic hyperpolarization, further diminishing the already reduced excitatory drive in peri-infarct networks. An emerging body of evidence also implicates adaptive immune-mediated mechanisms, with upregulated immune responses to brain antigens correlating with PSR onset - particularly when triggered by concurrent infection [7].

Critically, because these precipitants are transient, PSR follows a predictable course of resolution: symptoms lasted a mean of 18.4 hours in the largest retrospective cohort, with 69% resolving within 24 hours as systemic stressors were corrected and anesthetic concentrations declined, allowing marginal peri-infarct networks to resume their prior level of functional compensation [1]. 

Pharmacological evidence

Midazolam

The most direct human evidence linking a specific anesthetic agent to PSR comes from a prospective midazolam challenge study. The study administered intravenous midazolam to eight patients with image-verified stroke (occurring 7 days to 6 years prior) who had shown substantial clinical improvement from their initial syndromes [8].

Under conditions of light sedation, all five patients with left hemisphere stroke demonstrated reemergence or worsening of right hemiparesis and aphasia. All three patients with right hemisphere stroke showed reemergence of left hemiparesis and left visual field neglect - each patient's deficits faithfully recapitulated the topography of the original stroke syndrome [8]. All patients returned to baseline within two hours of drug clearance. A subsequent double-blinded crossover study by the same group confirmed these findings in 12 patients, demonstrating that midazolam - but not the anticholinergic agent scopolamine - re-induced prior motor and language deficits, providing beginning clinical evidence for the specificity of GABA(A)-sensitive pathways in stroke recovery [9]. These studies constitute the strongest direct evidence that a single pharmacologic agent can provoke PSR through a defined neurotransmitter mechanism. However, both studies involved small samples (8 and 12 patients, respectively) conducted under controlled experimental conditions, limiting generalizability to the heterogeneous perioperative environment.

Benzodiazepines

Beyond midazolam-specific challenge studies, the largest retrospective cohort of PSR (153 patients, 164 admissions) identified benzodiazepine exposure as significantly more common during PSR admissions compared with adjacent non-PSR admissions in the same patients [1]. However, concomitant illnesses and physiologic stressors, including infection, hypotension, hyponatremia, insomnia, or stress, were also present and represent potential confounding factors, limiting attribution of PSR specifically to benzodiazepine exposure [1].

Benzodiazepine use was classified alongside infection, hypotension, hyponatremia, and insomnia or stress as an important precipitant [1]. However, this study did not establish a specific benzodiazepine agent, dose, route, or causal relationship, as multiple triggers frequently co-occurred during PSR episodes [1].

A separate prospective study of 54 patients with carotid disease or brain tumors found that sedation with either midazolam or fentanyl produced focal motor deterioration in 30% of patients, with 73% of those who had a prior motor abnormality demonstrating exacerbation or unmasking of deficits [10]. While this study included patients with brain pathology beyond stroke, it further supports the vulnerability of previously injured neural networks to sedative-induced functional disruption.

Opioids and Other Anesthetics

Direct evidence linking individual opioid agents to PSR is weak. In the study by Thal et al., fentanyl unmasked focal motor deficits at rates comparable to midazolam [10]. Although the largest PSR cohort also cited prior clinical reports of symptom reemergence with fentanyl citrate, no controlled study has established opioids as independent PSR triggers in patients with prior stroke [1,10].

A systematic review of 12 studies identified reports of consistent recurrence of neurological deficits following administration of benzodiazepines and opioids, and noted that reversal with naloxone and flumazenil restored baseline function, but the review could not disentangle the individual contributions of specific agents [11]. Propofol, volatile anesthetics, and dexmedetomidine have not been adequately studied as independent PSR triggers. While preclinical data suggest potential neuroprotective properties for propofol and dexmedetomidine in acute ischemic stroke models, these investigations address acute neuroprotection rather than recrudescence of chronic deficits [12,13]. No clinical study has examined whether these agents provoke or protect against PSR. The available evidence regarding anesthetic, sedative, and analgesic agents potentially associated with PSR is summarized in Table 1.

**Table 1:** Evidence summary of anesthetic and sedative agents implicated in post-stroke recrudescence. This table summarizes the available human and preclinical evidence linking specific anesthetic, sedative, and analgesic agents to post-stroke recrudescence (PSR). The proposed mechanism, level of evidence, and key findings are presented for each agent class. Evidence strength ranges from controlled prospective challenge studies (midazolam) to indirect preclinical data (propofol, dexmedetomidine). GABA = gamma-aminobutyric acid; NMDA = N-methyl-D-aspartate; PSR = post-stroke recrudescence; NIHSS = National Institutes of Health Stroke Scale; AHA = American Heart Association; ASA = American Stroke Association.

Agent/class	Proposed mechanism	Study design	Sample size	Key findings	Level of evidence	References
Midazolam	GABA(A) agonism; augments pathologically elevated tonic inhibition via extrasynaptic α5- and δ-subunit-containing GABA(A) receptors in peri-infarct cortex	Prospective challenge study; double-blinded crossover trial	n = 8; n = 12	All patients demonstrated transient reemergence of prior stroke deficits (hemiparesis, aphasia, neglect) faithfully recapitulating original stroke topography; deficits resolved within 2 hours of drug clearance; midazolam but not scopolamine re-induced deficits	Strongest direct human evidence (small sample, controlled conditions)	Lazar et al. (2002) [8]; Lazar et al. (2010) [9]
Benzodiazepines	Broad GABA(A) subunit affinity enhances both tonic and phasic GABAergic currents; detrimental effect driven primarily by augmentation of pathologically elevated tonic inhibition	Retrospective crossover cohort and case-control study	n = 153 (164 PSR admissions)	Benzodiazepine exposure significantly more common during PSR admissions vs. adjacent non-PSR admissions; classified as an important precipitant alongside infection, hypotension, hyponatremia, and insomnia/stress	Moderate (retrospective; no specific agent, dose, or causal relationship established; multiple triggers co-occurred)	Topcuoglu et al. (2017) [1]
Fentanyl / Opioids	μ-receptor-mediated presynaptic inhibition of glutamate release and postsynaptic hyperpolarization; reduces excitatory drive in peri-infarct networks	Prospective observational study; retrospective cohort; systematic review	n = 54; n = 153; 12 studies reviewed	Fentanyl unmasked focal motor deficits at rates comparable to midazolam (30% of patients; 73% with prior motor abnormality); reversal with naloxone/flumazenil restored baseline; no controlled study establishes opioids as independent PSR triggers	Weak to moderate (mixed populations including brain tumors; unable to disentangle individual agent contributions)	Topcuoglu et al. (2017) [1]; Thal et al. (1996) [10]; Rizk et al. (2022) [11]
Propofol	GABA(A) receptor potentiation; preclinical data suggest possible neuroprotective properties (reduced oxidative injury, apoptotic signaling) in acute ischemic stroke models	Narrative review of preclinical and limited clinical data	N/A	Experimental studies show reduced oxidative injury and apoptotic signaling; no clinical study has examined whether propofol provokes or protects against PSR	Insufficient/Indirect (preclinical neuroprotection data only; not studied in recrudescence context)	Gandolfo et al. (2026) [12]
Volatile anesthetics	Multiple receptor targets including GABA(A) potentiation, NMDA antagonism, and two-pore potassium channel activation	Expert consensus /scientific statement	N/A	AHA/ASA concluded current evidence does not clearly characterize anesthetic agents as neuroprotective or neurotoxic; anesthetic choice unlikely to affect perioperative stroke risk (applies to de novo stroke, not PSR specifically)	Insufficient (no direct PSR data)	Benesch et al. (2021) [3] (AHA/ASA Scientific Statement)
Dexmedetomidine	α2-adrenergic agonism; preclinical data suggest attenuation of neuroinflammation and sympathetic overactivation in acute ischemic stroke models	Narrative review of preclinical and limited clinical data	N/A	Experimental studies show attenuation of neuroinflammation; no clinical study has examined whether dexmedetomidine provokes or protects against PSR	Insufficient/Indirect (preclinical neuroprotection data only; not studied in recrudescence context)	Gandolfo et al. (2026) [12]

General anesthesia

Isolating the contribution of any single anesthetic agent to PSR in the surgical setting is inherently difficult. General anesthesia entails simultaneous exposure to multiple potential triggers - including hypotension, hypoxemia, electrolyte abnormalities, systemic inflammation, pain, and sleep disruption - each of which has been independently associated with PSR [1]. The AHA/ASA Scientific Statement on perioperative stroke management concluded that current evidence does not clearly characterize anesthetic agents as either neuroprotective or neurotoxic [3]. It also stated that anesthetic choice is unlikely to affect perioperative stroke risk, although this conclusion applies to de novo stroke rather than post-stroke recrudescence [3]. The multifactorial nature of the perioperative environment therefore precludes attribution of PSR to the anesthetic medication itself without carefully controlled prospective studies.

Overall, the direct available evidence linking anesthetic agents to PSR remains limited and varies substantially by agent. The strongest human evidence involves midazolam and broader benzodiazepine exposure, whereas opioids have supportive but less definitive clinical evidence [1,8-11]. In contrast, evidence for propofol and dexmedetomidine is limited to indirect or preclinical studies of acute ischemic stroke rather than PSR [12].

The clinical response may also depend on medication dose, timing of administration, duration of exposure, and drug clearance. Future prospective studies should systematically document specific agents, doses, timing, perioperative stressors, and the temporal relationship between drug clearance and neurologic recovery, as available studies do not consistently report these variables [1,8-11].

Perioperative presentation and the literature gap

Despite the significant risk of diagnostic error, PSR remains unaddressed within existing perioperative neurological frameworks. While the AHA/ASA Scientific Statement recommends routine postanesthesia neurological assessments for high-risk patients and provides structured approaches to perioperative stroke risk stratification, none explicitly distinguish recrudescence of prior deficits from de novo ischemic injury or residual anesthetic effect [3,14]. This diagnostic gap is clinically consequential.

This gap precipitates potentially avoidable cascade effects. These include redundant emergency neuroimaging that diverts resources and delays definitive care; prolonged postanesthesia care unit stays that independently increase the risk of postoperative delirium, in-hospital falls, and 30-day mortality [15]; and, in rare instances, the administration of thrombolytic therapy for a non-ischemic etiology-a scenario documented in 4% of PSR cases-which poses avoidable hemorrhagic risk [1]. 

Incorporating PSR as a distinct diagnostic entity into perioperative neurological assessment algorithms is therefore essential for accurate triage, patient safety, and resource management.

Diagnostic challenges in the postoperative period

Distinguishing post-stroke recrudescence from other postoperative neurologic complications requires a structured approach emphasizing clinical history and symptom onset [16]. A defining feature of PSR is that focal deficits mirror the patient’s prior stroke manifestations, whereas new perioperative stroke or transient ischemic attacks typically present with novel findings [17]. Unlike acute ischemic injury, which involves new diffusion-weighted imaging lesions, PSR is characterized by the absence of fresh radiologic infarcts and rapid clinical improvement upon mitigation of systemic stressors or clearance of anesthetic agents [18]. Furthermore, differentiating PSR from seizures, delirium, or residual anesthetic effects requires careful observation of consciousness and EEG activity; PSR typically manifests as isolated focal deficits in the absence of global cognitive clouding or epileptiform discharges. A 2023 prospective single-center Chinese cohort reinforced these distinctions, identifying PSR in 2.4% of suspected strokes and showing that PSR - unlike TIA - was frequently precipitated by infection (22.2% vs 2%) and associated with worse 3-month functional independence (80% vs 98%) and less early neurological improvement, with deficits resolving over several days once the trigger was corrected [19]. The key features distinguishing PSR from acute perioperative ischemic stroke, seizure or Todd paralysis, postoperative delirium, and residual anesthetic effects are summarized in Table 2.

**Table 2:** Perioperative differential diagnosis; post-stroke recrudescence versus acute ischemic stroke, seizure/postictal state, postoperative delirium, and residual anesthetic effect. This table compares the key clinical, radiologic, and electroencephalographic features that distinguish post-stroke recrudescence (PSR) from its principal perioperative mimics. PSR is characterized by topographic fidelity to the prior stroke syndrome, absence of new diffusion-weighted imaging (DWI) abnormalities, and resolution upon correction of the precipitating trigger. PSR remains a diagnosis of exclusion; its consideration should not delay evaluation for acute ischemic stroke. DWI = diffusion-weighted imaging; EEG = electroencephalography; NIHSS = National Institutes of Health Stroke Scale; PACU = post-anesthesia care unit.

Feature	Post-stroke recrudescence	Acute perioperative ischemic stroke	Seizure/Todd paralysis	Postoperative delirium	Residual anesthetic effect	References
Onset	Usually abrupt; may initially progress	Abrupt; onset often obscured by anesthesia or sedation	Abrupt; associated with seizure activity; maximum at onset	Acute to subacute; fluctuating course	Gradual emergence; temporally linked to anesthesia offset	[1,3,17]
Deficit pattern	Focal deficits that mirror prior stroke topography (motor, sensory, language); a subset of previous stroke symptoms; no isolated gaze paresis, hemianopia, or neglect	Novel focal deficits; may not correspond to prior stroke territory; anterior circulation predominance	Focal deficits that may extend beyond original stroke symptoms; may include confusion, tongue bite, incontinence	Global cognitive clouding; inattention; disorientation; non-focal; fluctuating	Global: sedation, cognitive slowing, non-focal; symmetric motor depression	[1,8,16,17]
Severity	Mild to moderate; NIHSS worsening mean 2.5 points; does not exceed prior stroke deficits	Variable; often moderate to severe; >50% have severe disability	Variable; may be severe	Variable; typically non-focal	Variable; dose-dependent	[1,17,18]
DWI on MRI	No new diffusion restriction; chronic infarct present	New acute diffusion-restricted lesion(s)	Usually normal; may be abnormal if seizure is prolonged	No new acute infarct (unless concurrent stroke)	No new acute infarct	[1,17,18]
EEG findings	No epileptiform discharges	No epileptiform discharges (unless concurrent seizure)	Ictal or postictal epileptic/epileptiform patterns	May show diffuse slowing; no epileptiform discharges	Diffuse slowing consistent with residual anesthetic effect	[1,4,16]
Identifiable trigger	Systemic stressor: infection, hypotension, hyponatremia, benzodiazepine/opioid exposure, insomnia/stress	Thromboembolism, hypoperfusion, arrhythmia (e.g., atrial fibrillation), vascular manipulation	Epileptogenic focus; metabolic derangement; medication withdrawal	Pain, infection, metabolic derangement, anticholinergic medications, sleep disruption	Residual drug effect; prolonged emergence	[1,3,15,17]
Time course	Mean duration 18.4 hours; 69% resolve within 24 hours; resolves with trigger correction or drug clearance	Persistent unless treated; may worsen without intervention	Todd paralysis: resolves within minutes to hours (usually <48 h)	Hours to days; fluctuating; resolves with treatment of underlying cause	Resolves with drug metabolism; typically minutes to hours	[1,11,17]
Response to reversal agents	Improvement with flumazenil (benzodiazepines) or naloxone (opioids) when these agents are the trigger	No response to reversal agents; requires reperfusion therapy	Responds to antiseizure medications	May respond to treatment of underlying cause; haloperidol or other agents for symptom management	Responds to specific reversal agents (e.g., sugammadex, flumazenil, naloxone)	[1,3,11]
Key distinguishing principle	Diagnosis of exclusion; topographic fidelity to prior stroke syndrome; absence of new ischemic lesion	New vascular territory involvement; DWI-positive lesion; vascular occlusion on angiography	Epileptiform activity on EEG; positive symptoms (e.g., clonic movements) may precede deficit	Global, non-focal cognitive disturbance; fluctuating attention and arousal	Temporal relationship to anesthesia; symmetric, non-focal; resolves predictably with time	[1,3,16,18]

Cognitive biases and specialty silos in perioperative care

Diagnostic reasoning in the perioperative environment is frequently hampered by anchoring bias, a cognitive error implicated in 36.5 to 77% of diagnostic inaccuracies [20]. This susceptibility, which affects both general practitioners and specialists, leads clinicians to prematurely solidify diagnoses such as new ischemic stroke or postoperative surgical complication without considering transient metabolic or pharmacological mimics.

This cognitive rigidity is exacerbated by specialty-based compartmentalization; fragmented communication pathways between anesthesiologists, surgeons, and neurologists hinder the integration of medication history with clinical observation, a known driver of diagnostic delay [21]. This lack of interdisciplinary synergy leads to redundant neuroimaging and burdensome diagnostic workups that delay the recognition of reversible anesthetic effects [22]. Consequently, this procedural fragmentation precludes the timely consideration of corrective measures-such as the strategic administration of reversal agents or the prioritization of expedited drug clearance-thereby unnecessarily prolonging symptomatic manifestation. Such diagnostic inertia commits the patient to burdensome testing and potential inappropriate escalation of care.

Clinical implications and recommendations

To mitigate the risk of diagnostic misclassification, perioperative teams should consider incorporating several practical measures into routine practice. Mandatory preoperative screening should document each patient's specific baseline neurologic deficits and prior stroke history; notably, no standardized preoperative neurological screening protocol currently exists for surgical patients with cerebrovascular disease, and the AHA/ASA has acknowledged that no stroke assessment scale has been validated in the perioperative setting [3].

When sudden postoperative focal neurologic deficits occur, clinicians should immediately initiate an acute stroke evaluation while comparing the findings with the documented preoperative baseline and prior stroke syndrome. Prompt neurologic consultation and appropriate neuroimaging are warranted to evaluate for new ischemia, hemorrhage, seizure, and other potential causes. PSR should be considered when the deficits reproduce the patient’s prior stroke pattern and no new acute lesion is identified [23]; however, its consideration should not delay time-sensitive stroke evaluation or treatment. Once acute stroke has been excluded, recognition of PSR may prevent unnecessary additional testing and inappropriate interventions.

## Conclusions

Post-stroke recrudescence is an under-recognized but clinically important stroke mimic in the perioperative setting. Anesthetic and analgesic agents, particularly benzodiazepines and opioids, can unmask latent deficits by disrupting fragile peri-infarct networks that depend on carefully balanced neuroplasticity. Recognition of the characteristic topographic fidelity of deficits, absence of new diffusion restriction, and rapid resolution after trigger correction enables accurate diagnosis. Addressing cognitive biases and specialty silos through structured preoperative documentation and interdisciplinary communication will further reduce diagnostic error, prevent unnecessary interventions, and improve resource utilization. Greater emphasis on PSR within anesthesia and perioperative guidelines is warranted.
